# Phase-dependent oscillatory dynamics during needle retention and withdrawal: a comparison of penetrating and non-penetrating acupuncture

**DOI:** 10.3389/fpain.2026.1842608

**Published:** 2026-09-23

**Authors:** Qian Wu, Dehui Lin, Shanze Wang, Xuesong Liang, Bingxin Wu, Qing Yang, Fangyuan Zhang, Xiuyun Wen, Wenbin Fu

**Affiliations:** 1 Department of Acupuncture and Moxibustion, The Second Affiliated Hospital of Guangzhou University of Chinese Medicine (Guangdong Provincial Hospital of Chinese Medicine), Guangzhou, China; 2 School of Traditional Chinese Medicine, Guangdong Pharmaceutical University, Guangzhou, China; 3 Traditional Chinese Medicine Department, Foshan Sanshui District People's Hospital, Foshan, China; 4 School of Health Science, Guangdong Pharmaceutical University, Guangzhou, China; 5 Innovative Research Team of Acupuncture for Depression and Related Disorders, The Second Affiliated Hospital of Guangzhou University of Chinese Medicine (Guangdong Provincial Hospital of Chinese Medicine), Guangzhou, China

**Keywords:** band power, electroencephalography, non-penetrating, non-specific effects, sham acupuncture

## Abstract

**Introduction:**

Clinical and neuroimaging studies have shown that non-penetrating sham acupuncture can elicit effects comparable to those of verum acupuncture. However, the electrophysiological signatures on the scalp remain unclear.

**Method:**

In a 2 × 2 crossover study involving 20 acupuncture-naive college students, we recorded electroencephalography (EEG) during verum and sham acupuncture at Hegu (LI4) to determine whether sham acupuncture evokes scalp electrophysiological activities comparable to verum acupuncture during the needle retention and post-withdrawal phases.

**Results:**

Despite eliciting a stronger deqi sensation, verum acupuncture produced scalp electrophysiological responses that closely resembled those evoked by sham acupuncture. Both interventions were associated with a significant increase in theta power in the central region during the needle retention and post-withdrawal phases, a significant increase in beta power in the central region during the needle retention phase, and a widespread reduction in alpha power from the needle retention to the post-withdrawal phase. Nevertheless, sham acupuncture may exert a relatively weaker influence, and the most consistent difference between verum and sham acupuncture concerned the alpha power reduction from the needle retention phase to the post-withdrawal phase. Exploratory correlation analyses indicated that the associations between deqi sensations and scalp EEG power may differ between verum and sham acupuncture.

**Conclusion:**

These results indicate that non-penetrating skin touch stimulation at acupoints and the associated sensory attention can trigger electrophysiological activities similar to, though partially weaker than, those of verum acupuncture.

## Introduction

A persistent challenge in acupuncture research is that: despite its substantial effectiveness in clinical practice, randomized controlled trials (RCTs) frequently report only modest advantages over placebo acupuncture controls, particularly for pain and emotional disorders (1–3). These observations have fueled stigmatizing assertions that acupuncture is “nothing more than placebo”, contributing to a so-called “placebo stalemate” in the field. Some scholars contend that this impasse reflects limitations in placebo-control design rather than an inherent failure of acupuncture as an intervention. To better isolate the specific effects of acupuncture, researchers have made extensive efforts to refine placebo acupuncture control designs (4, 5). However, for a complex intervention such as acupuncture, separating non-specific effects remains challenging (6). Notably, some effects labeled as “non-specific” may in fact represent intrinsic components of acupuncture therapy (7).

In recent decades, non-penetrating tactile stimulation at acupoints has been widely used in RCTs to maximize blinding while minimizing physical impact. Nevertheless, the field has not yet escaped the “placebo stalemate”. Researchers have gradually recognized that most placebo acupuncture procedures are not physiologically inert and have suggested that this control be termed “sham acupuncture” (8). It is increasingly recognized that the effects elicited by sham acupuncture may reflect complex cognitive and affective processes (9, 10). Recent studies have provided important impetus to investigate the psychological effects of acupuncture. We found that both verum and sham acupuncture interventions can rapidly induce similar emotional reactivity and electrophysiological regulation (11). The psychological components of sham acupuncture may extend beyond a placebo response and include, among other factors, non-penetrating surface stimulation, attentional focus, and related psychological regulation. The therapeutic components common to both verum and sham acupuncture contribute to the observed efficacy, a fact that leads to a significant underestimation of the true treatment effect of verum acupuncture.

Prior mechanistic studies of acupuncture have emphasized immediate changes of post verum treatment relative to baseline. Recent electroencephalogram (EEG) studies have even provided increasingly sophisticated methods including deep learning to characterize the brain activity related to acupuncture (12–15). However, studies exploring the effects of non-penetrating acupuncture on electrophysiological activities are still quite scarce. Notably, an early meta-analysis reported that the use of von Frey filaments or non-penetrating sham acupuncture induces a weaker BOLD response pattern in brain regions similar to those activated by verum acupuncture (16). Recent studies on phantom acupuncture (17) and acupuncture imagery (18) further suggest that, even in the complete absence of somatosensory stimulation, deliberate visual stimulation and acupuncture-related imagery can evoke electrophysiological activity resembling that elicited by verum acupuncture. However, we propose that even in the absence of deliberate imagination or visual stimulation, non-penetrating tactile stimulation at acupoints — together with the accompanying sensory attention — may be sufficient to evoke electrophysiological activity comparable to that induced by verum acupuncture, and this might serve as the basis for its clinical therapeutic effect.

The aim of the current study was to compare the acute, phase-dependent scalp electrophysiological activities induced by non-penetrating sham acupuncture and verum acupuncture at Hegu (LI4) acupuncture point. LI4 was selected as the stimulation site due to its widespread clinical use for pain and emotional disorders and its central role in the treatment protocol currently being investigated by our team (19). Furthermore, individuals may exhibit relatively high sensory discriminability for stimulation applied to LI4 (20). To minimize confounding influences related to expected clinical efficacy, we recruited healthy participants. Following baseline assessment, participants underwent verum and sham acupuncture in a counterbalanced, random order. Participants were instructed to close their eyes, remain relaxed, and attend to the somatosensory sensations evoked by acupuncture. To better characterize the effects of these procedures, we propose shifting the temporal focus away from the brief, artifact-prone manipulation phase, and toward the clinically meaningful yet underexplored phases of static stimulation (retention) and post-stimulation (withdrawal). In clinical practice, the needle retention phase typically lasts far longer than the manipulation phase and is of substantial clinical relevance (21, 22). The retention phase offers a unique opportunity to examine acupuncture-evoked psychological regulation, while the ongoing low-intensity sensory input may serve as a substrate for specific neural pathways. More critically, the post-manipulation phase may reveal unique after-effects, during which differences between verum and sham acupuncture may still be detectable. The classic electroencephalogram (EEG) band power reflects the synchronous neural discharging and has strong functional interpretability. Changes in EEG power can reflect shifts in arousal, affective state, and cognitive processing. Finally, to probe the potential drivers of any observed effects, we assessed the association between EEG power and participants' subjective experience of deqi.

## Materials and methods

### Participants

The sample comprised 20 right-handed, acupuncture-naive college students (15 males), aged 18–24. Participants were screened and excluded for neurological, psychiatric, or medical disorders; use of conventional or alternative medications; head trauma with loss of consciousness; or any contraindications for acupuncture. All participants read and provided written informed consent.

### Procedure

The investigation was conducted at Sun Yat-sen University. Participants were seated comfortably in a dimly lit, sound-attenuated room. The experiment began with a baseline period of eyes-closed rest. Subsequently, participants were informed that acupuncture would be performed using two different stimulation techniques sequentially on the LI4 acupoint of their left hand. They were instructed to remain relaxed and to direct their attention on the sensations experienced during needling. Verum and sham acupuncture were delivered across two intervention sessions in a randomized, counterbalanced order, with a 30-minute (23–25) washout interval between sessions. The washout interval was chosen to keep the two stimulation sessions within a comparable experimental context, avoid removing and reapplying the EEG cap, and allow a reasonable interval for subjective sensations from the first stimulation to dissipate. Before the second intervention session, all participants reported that no residual sensation from the first session persisted in the left hand.

### Intervention

To minimize variability in participants' physical and mental states, each participant completed the entire experiment within a single day. To standardize the protocol duration, we referenced the “Qianjin Fang” and set the needle retention time to 5 min (21), with an equivalent recording period following needle withdrawal. As shown in Figure 1, each intervention session comprised three successive phases administered by an experienced acupuncturist: 1-minute needle manipulation, 5-minute needle retention (referred to as retention), and 5-minute post-withdrawal (referred to as withdrawal). A device resembling a plastic tube, similar to the “Park” device (26), was used to maintain participant blinding throughout the procedure, and participants kept their eyes closed. During verum acupuncture, a single-use sterile Huatuo acupuncture needle (pure silver, 0.25 mm diameter, 40 mm length) was inserted to a depth of 1.5–2 cm at the left Hegu acupoint. During the 1-minute manipulation phase, the needle was rotated bidirectionally with an even motion at an amplitude of approximately 180 degrees and a frequency of 1 Hz. During sham acupuncture, a blunt needle was applied without skin penetration, and rotational manipulation was not employed. Instead, the acupuncturist mimicked manipulation by gently tapping the needle handle at a frequency of 1 Hz during the 1-minute manipulation phase. At the conclusion of each intervention session, participants completed the Chinese version of the Massachusetts General Hospital Acupuncture Sensation Scale (MASS) questionnaire (27, 28). The MASS Index (MI), quantifying deqi intensity, was subsequently calculated using the 12 descriptors included in the MASS.

**Figure 1:**
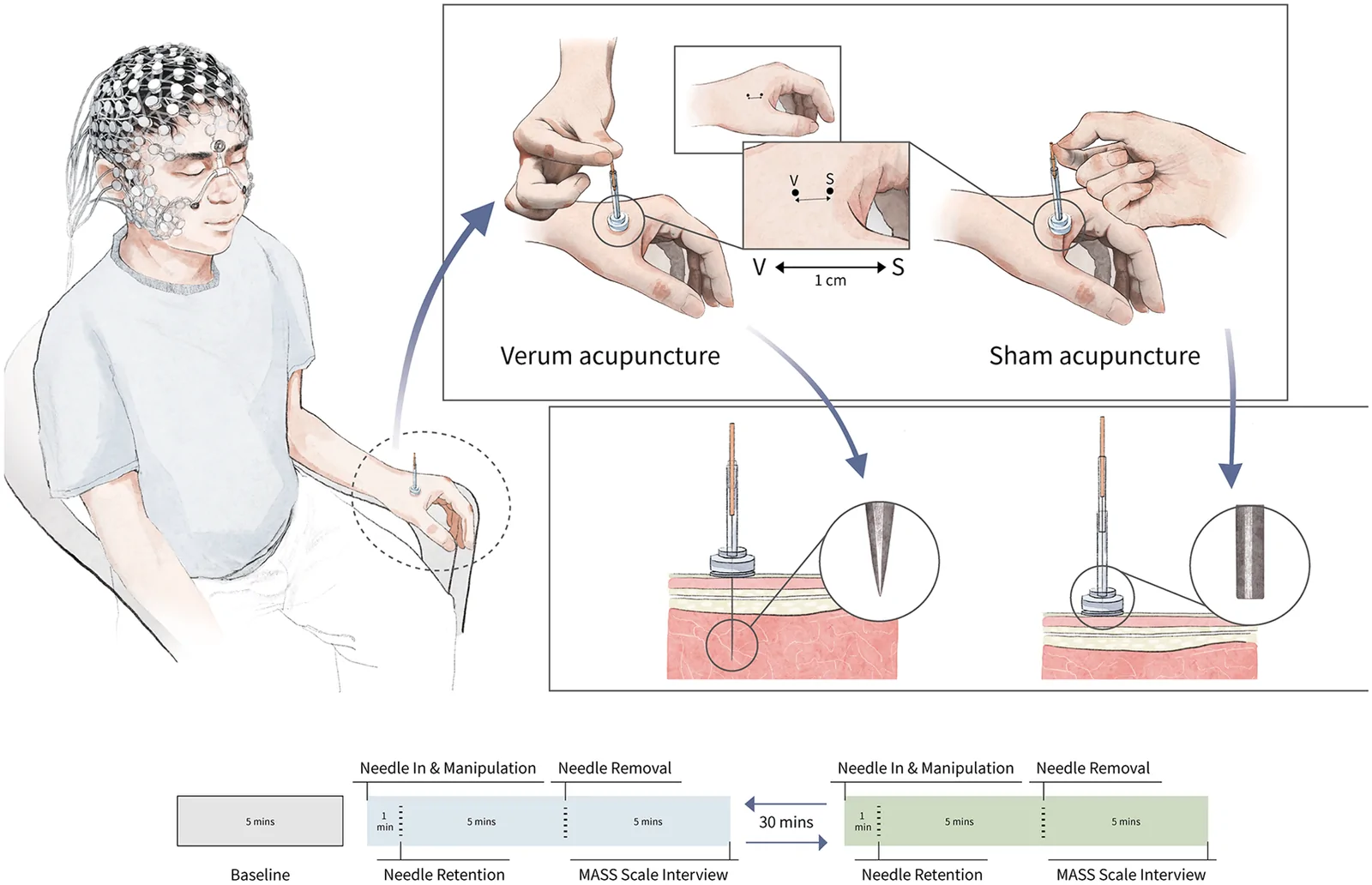
Experiment procedure and intervention schematic diagram.

### EEG acquisition and analysis

Resting-state EEG was recorded continuously throughout all experimental phases except the washout session using a 128-channel HydroCel Geodesic Sensor Net connected to a Net Amps 400 amplifier and Net Station Acquisition software (Electrical Geodesic, Inc., Eugene, Oregon, USA). Data were acquired at a sampling rate of 1,000 Hz with Cz serving as the online reference. Electrode impedance was maintained below 50 k*Ω* using a potassium chloride solution. Continuous EEG data were preprocessed through an automated pipeline utilizing MATLAB R2020b (The MathWorks, Natick, MA, USA) and EEGLAB 2022.1. The data were down-sampled to 100 Hz and filtered with 0.5 Hz high-pass and 47 Hz low-pass finite impulse response (FIR) filters. Bad channels were identified and rejected based on spectral characteristics and flat-line criteria. Artifact bursts were corrected using Artifact Subspace Reconstruction (29). Rejected channels were interpolated using spherical spline interpolation. Infomax Independent Component Analysis (ICA) was applied to remove artifact components, including ocular and muscle artifacts. Data quality was further verified through manual inspection. The cleaned EEG data were then re-referenced to the average reference. Power spectral density was computed for each EEG channel using Welch's method with 2-second Hanning windows. Based on previous research, the theta (4–8 Hz), alpha (8–12 Hz), and beta (12–30 Hz) frequency bands were defined (17, 30), and the absolute power (AP) was calculated. These electrophysiological indicators have been associated with internal monitoring, cortical idling or inhibitory processes, and sustained somatosensory attention (31, 32). As shown in Figure 2, AP values were averaged across channels within regions of interest (ROIs): anterior, central, posterior, left, and right regions, followed by natural logarithm transformation for subsequent analysis. To show the evolution of scalp electrophysiological activities during verum and sham acupuncture, time-frequency analysis was conducted using multi-taper method. The number of cycles in the wavelet was set to half of the frequency, and the power from 1 to 30 Hz is calculated with a frequency resolution of 1 Hz. Retention and post-withdrawal phases were baseline-corrected using the average power during the baseline period according to the gain model. Time-frequency data were averaged across channels within ROI. Based on the representativeness of the main results, the time–frequency data for the central and anterior regions are presented.

**Figure 2:**
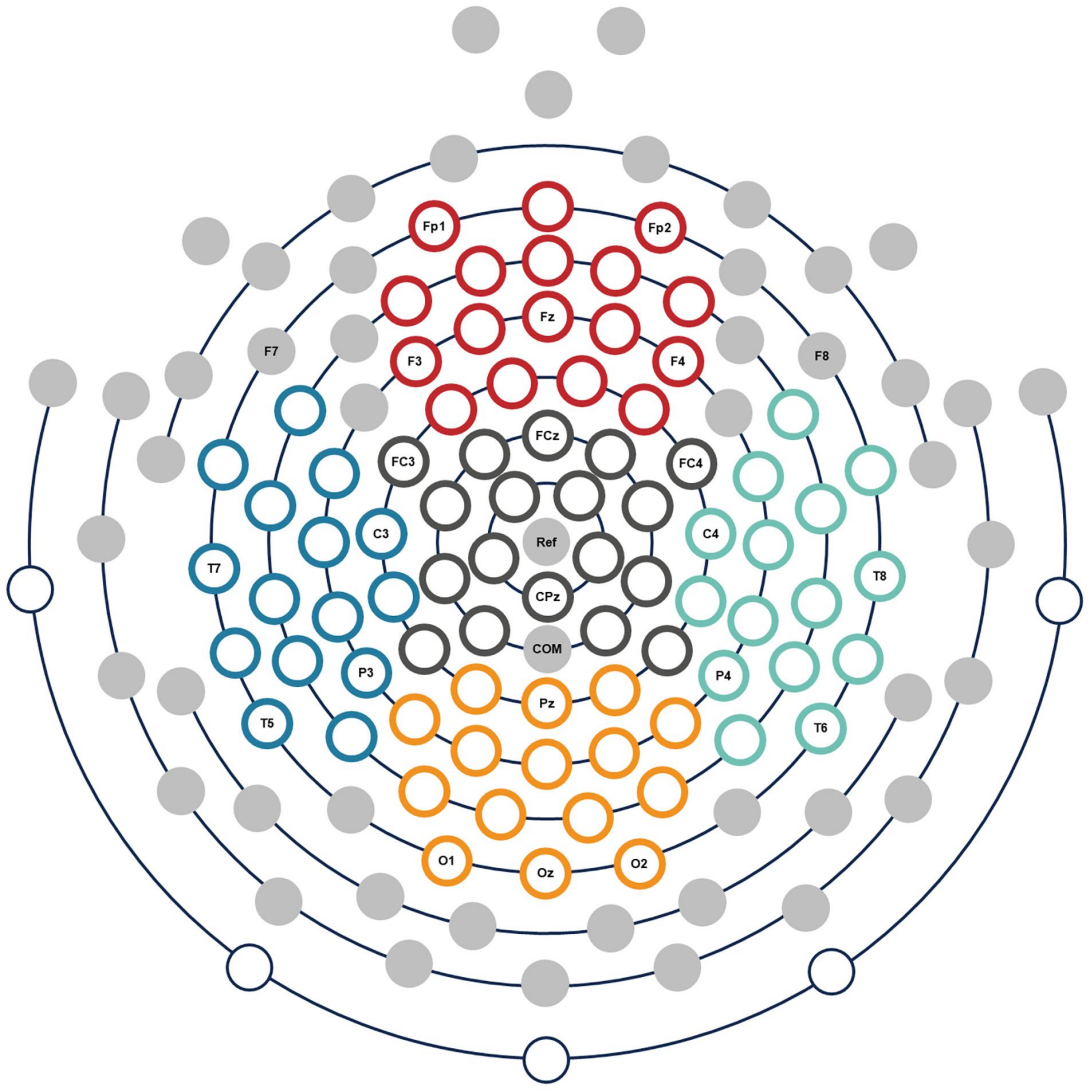
Hydrocel geodesic sensor Net-128 channel map. Red: anterior region; Black: central region; Orange: posterior region; Blue: left region; Green: right region.

### Statistical analysis

A one-way repeated-measures ANOVA (rm-ANOVA) with the factor intervention (verum vs. sham) was performed on the MI. Separate one-way rm-ANOVAs with the factor condition (five levels: baseline, verum retention, verum withdrawal, sham retention, sham withdrawal) were conducted for AP within each ROI. Degrees of freedom were adjusted using the Greenhouse-Geisser correction when appropriate. The 95% confidence intervals for the least squares means (LS-means) were derived from the rm-ANOVA models. Pearson's correlation coefficient was used to analyze the relationship between MI and AP.

## Result

### Sham acupuncture induced weaker deqi

Verum acupuncture [mean MI: 4.7; 95% CI: (3.5, 5.8)] elicited significantly stronger deqi sensation than sham acupuncture [mean MI: 2.7; 95% CI: (1.9, 3.5)], as measured by the MI [F (1, 19) = 8.65, *P* = 0.008, $\eta_{p}^{2}$  = 0.313]. As shown in Figure 3, these findings indicate stronger self-reported somatic sensations during verum acupuncture. Despite the difference, sham acupuncture still elicited measurable deqi-related sensations, albeit with less intense.

**Figure 3:**
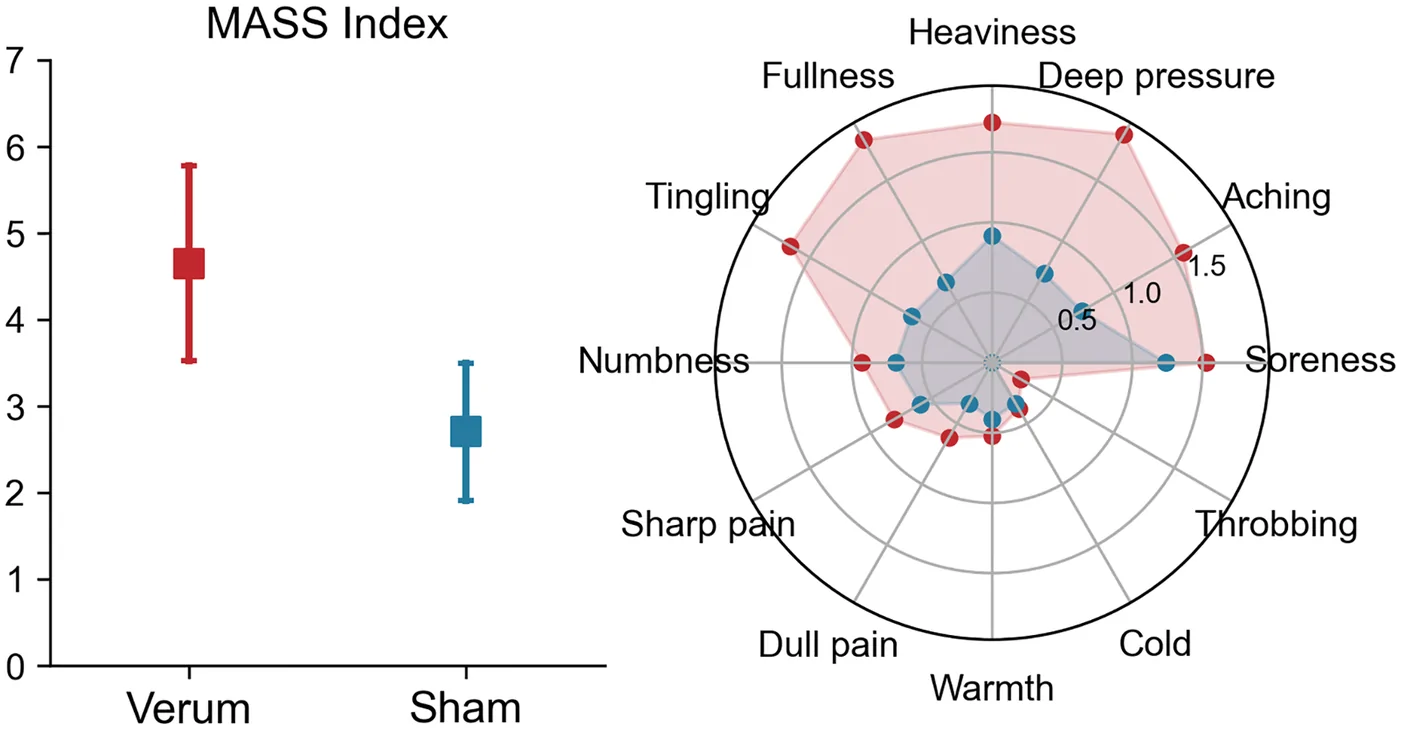
Deqi sensation induced by verum and sham acupuncture. Left, mean and 95% CI of deqi Index; Right, radar chart of the average values of all the MASS items.

### Both interventions increased theta power in central

A significant main effect of condition was observed in the central region [F(4, 76) = 3.54, *P* = 0.011, $\eta_{p}^{2}$  = 0.157]. Compared to baseline, theta power increased significantly during verum retention [mean difference=0.146, 95% CI: (0.051, 0.241), *P* = 0.005, Cohen's d = 0.350], verum withdrawal [mean difference = 0.187, 95% CI: (0.051, 0.323), *P* = 0.010, Cohen's d = 0.449], sham retention [mean difference = 0.114, 95% CI: (0.013, 0.216), *P* = 0.029, Cohen's d = 0.274], and sham withdrawal [difference = 0.138, 95% CI: (0.022, 0.254), *P* = 0.022, Cohen's d = 0.331]. No significant differences in theta power were observed between verum and sham acupuncture at any time point. Figure 4 and Table 1 provide a descriptive summary of these results.

**Figure 4:**
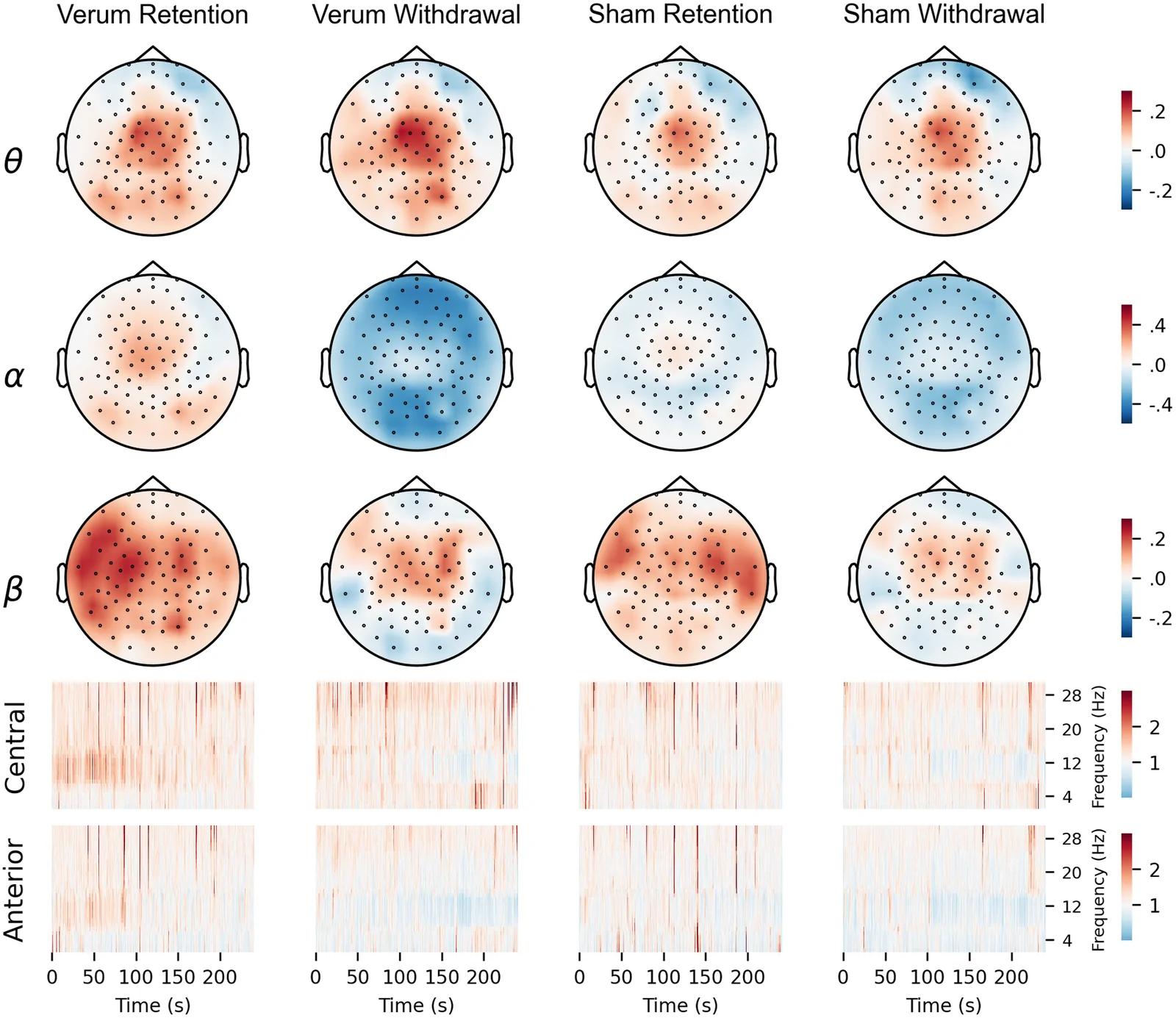
Power changes induced by verum and sham acupuncture. Top, grand-average scalp topographical maps of changes in natural log-transformed absolute power during intervention sessions relative to baseline; Bottom, time-frequency with baseline corrected following the gain model.

**Table 1:** Summary of power changes induced by verum and sham acupuncture.

Intervention	contrast	anterior^a^	central	posterior	left	right
Verum	Retention vs. baseline		*θ*↑, *β*↑		*β*↑	
	Withdrawal vs. baseline	*α*↓	*θ*↑, *β*↑			
	Withdrawal vs. retention	α↓↓	α↓↓	α↓↓	α↓↓, *β*↓	α↓↓
Sham	Retention vs. baseline		*θ*↑, β↑			
	Withdrawal vs. baseline	α↓	*θ*↑			
	Withdrawal vs. retention	α↓	α↓	α↓	β↓	α↓

a Single-arrow symbol indicates significant within-condition changes for the specified contrast, double-arrow symbol indicates a greater change relative to the other intervention, and blank cell indicates that no significant difference was observed.

### Both interventions reduced alpha power during withdrawal

Significant differences between conditions were observed in the anterior [F(1.86, 33.42) = 4.46, *P* = 0.021, $\eta_{p}^{2}$  = 0.190], central [F(2.00, 38.00) = 3.31, *P* = 0.047, $\eta_{p}^{2}$  = 0.149], posterior [F(2.13, 40.41) = 3.46, *P* = 0.038, $\eta_{p}^{2}$  = 0.154], left [F(2.08, 39.43) = 3.45, *P* = 0.040, $\eta_{p}^{2}$  = 0.154] and right [F(1.97, 37.50) = 3.61, *P* = 0.037, $\eta_{p}^{2}$  = 0.160] regions. Significant decreases from baseline were only found in the anterior region, where both verum withdrawal [mean difference = −0.361, 95% CI: (−0.722, −0.001), *P* = 0.050, Cohen's d = −0.355] and sham withdrawal [mean difference = −0.210, 95% CI: (−0.405, −0.016), *P* = 0.035, Cohen's d = −0.207] showed reduced alpha power.

For both interventions, we observed significant decreases from the retention to the withdrawal phase across all ROIs. For verum acupuncture, decreases were observed in anterior [mean difference = −0.421, 95% CI: (−0.618, −0.225), *P* < 0.001, Cohen's d = −0.414], central [mean difference = −0.351, 95% CI: (−0.515, −0.188), *P* < 0.001, Cohen's d = −0.375], posterior [mean difference = −0.433, 95% CI: (−0.649, −0.217), *P* *<* 0.001, Cohen's d = −0.381], left [mean difference = −0.337, 95% CI: (−0.508, −0.165), *P* *<* 0.001, Cohen's d = −0.385], and right [mean difference = −0.337, 95% CI: (−0.509, −0.164), *P* *<* 0.001, Cohen's d = −0.361] regions. For sham acupuncture, decreases were found in anterior [mean difference = −0.160, 95% CI: (−0.288, −0.032), *P* *=* 0.017, Cohen's d = −0.158], central [mean difference = −0.132, 95% CI: (−0.253, −0.011), *P* *=* 0.035, Cohen's d = −0.141], posterior [mean difference = −0.170, 95% CI: (−0.323, −0.017), *P* *=* 0.031, Cohen's d = −0.150], and right [mean difference=−0.117, 95% CI: (−0.229, −0.005), *P* *=* 0.042, Cohen's d = −0.125] regions, with a non-significant trend in the left region [mean difference=−0.116, 95% CI: (−0.246, 0.013), *P* *=* 0.075, Cohen's d = −0.133]. Linear contrast tests revealed that the decreases in alpha power were significantly larger for verum than sham acupuncture across all regions: anterior [mean difference = −0.261, 95% CI: (−0.480, −0.042), *P* *=* 0.022, Cohen's d = 0.257], central [mean difference = −0.219, 95% CI: (−0.415, −0.024), *P* *=* 0.030, Cohen's d = 0.234], posterior [mean difference=−0.263, 95% CI: (−0.510, −0.015), *P* *=* 0.039, Cohen's d = 0.231], left [mean difference = −0.220, 95% CI: (−0.409, −0.031), *P* *=* 0.025, Cohen's d = 0.252], and right [mean difference = −0.220, 95% CI: (−0.414, −0.026), *P* *=* 0.029, Cohen's d = 0.236]. Figure 4 and Table 1 provide a descriptive summary of these results.

### Both interventions increase Beta power in central

Significant differences between conditions were observed in the central [F(2.63, 49.90) = 3.64, *P* = 0.023, $\eta_{p}^{2}$  = 0.161] and left [F(4, 76) = 4.32, *P* = 0.003, $\eta_{p}^{2}$  = 0.185] regions. In the central region, beta power increased from baseline during verum retention [mean difference = 0.166, 95% CI (0.071, 0.452), *P* = 0.002, Cohen's d = 0.272], verum withdrawal [mean difference = 0.139, 95% CI (0.021, 0.257), *P* = 0.023, Cohen's d = 0.229], sham retention [mean difference = 0.118, 95% CI (0.039, 0.198), *P* = 0.006, Cohen's d = 0.194], and sham withdrawal [mean difference = 0.082, 95% CI (−0.010, 0.173), *P* = 0.078, Cohen's d = 0.134]. No significant differences were found between verum and sham acupuncture. In the left region, beta power during verum retention was significantly higher than at baseline [mean difference = 0.198, 95% CI (0.046, 0.350), *P* = 0.014, Cohen's d = 0.346] and also significantly higher than during sham retention [mean difference = 0.115, 95% CI (0.006, 0.223), *P* = 0.040, Cohen's d = 0.200]. Additionally, both verum [mean difference = −0.192, 95% CI (−0.296, −0.088), *P* = 0.001, Cohen's d = −0.336] and sham [mean difference = −0.102, 95% CI (−0.203, 0.000), *P* = 0.050, Cohen's d = −0.177] acupuncture showed significant decreases in beta power from the retention to withdrawal phase. Figure 4 and Table 1 provide a descriptive summary of these results.

### Different correlations between AP and MI between Verum and sham acupuncture

We examined the association between the MI and AP. As shown in Figures 5, 6, MI was significantly negatively correlated with theta AP in the verum acupuncture condition, whereas it was significantly positively correlated with beta AP in the sham acupuncture condition. No significant correlation was observed between alpha AP and MI.

**Figure 5:**
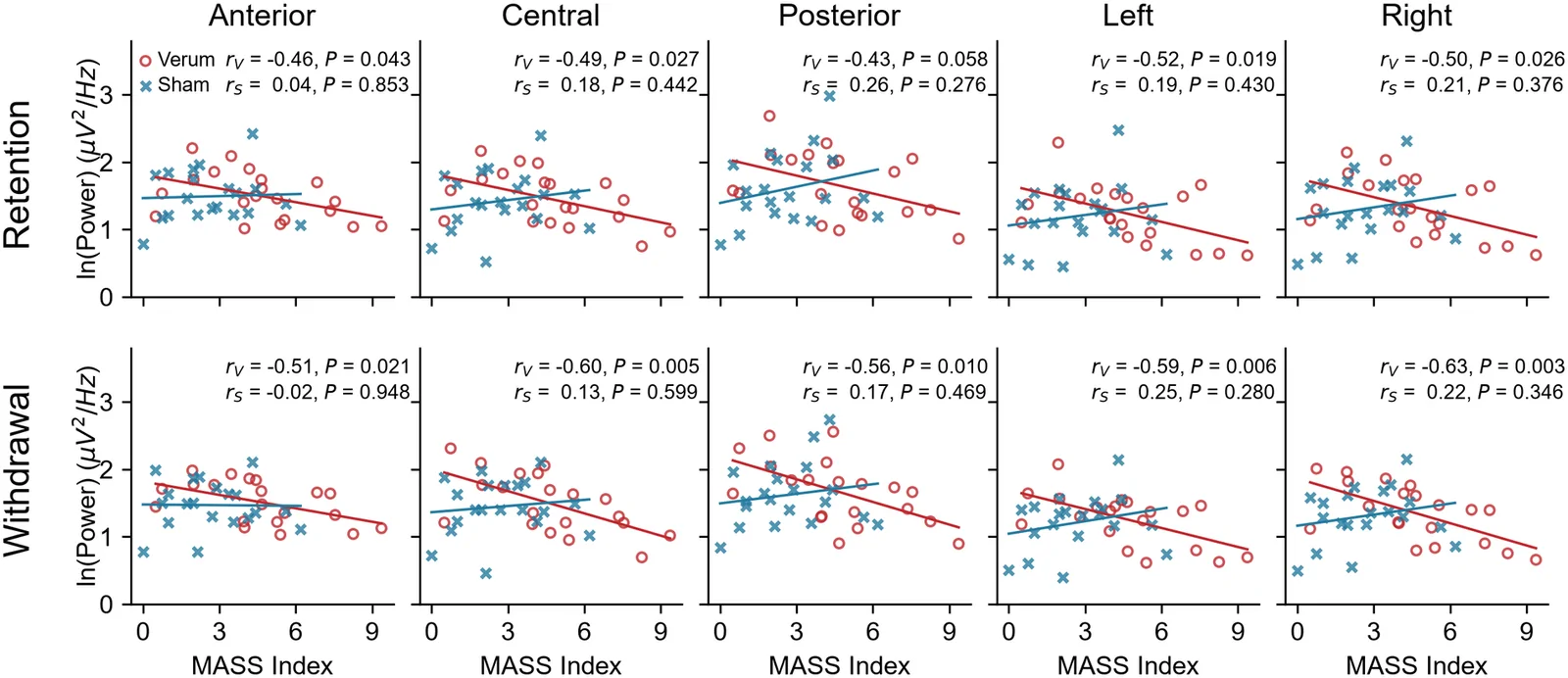
Correlation between MI and theta AP.

**Figure 6:**
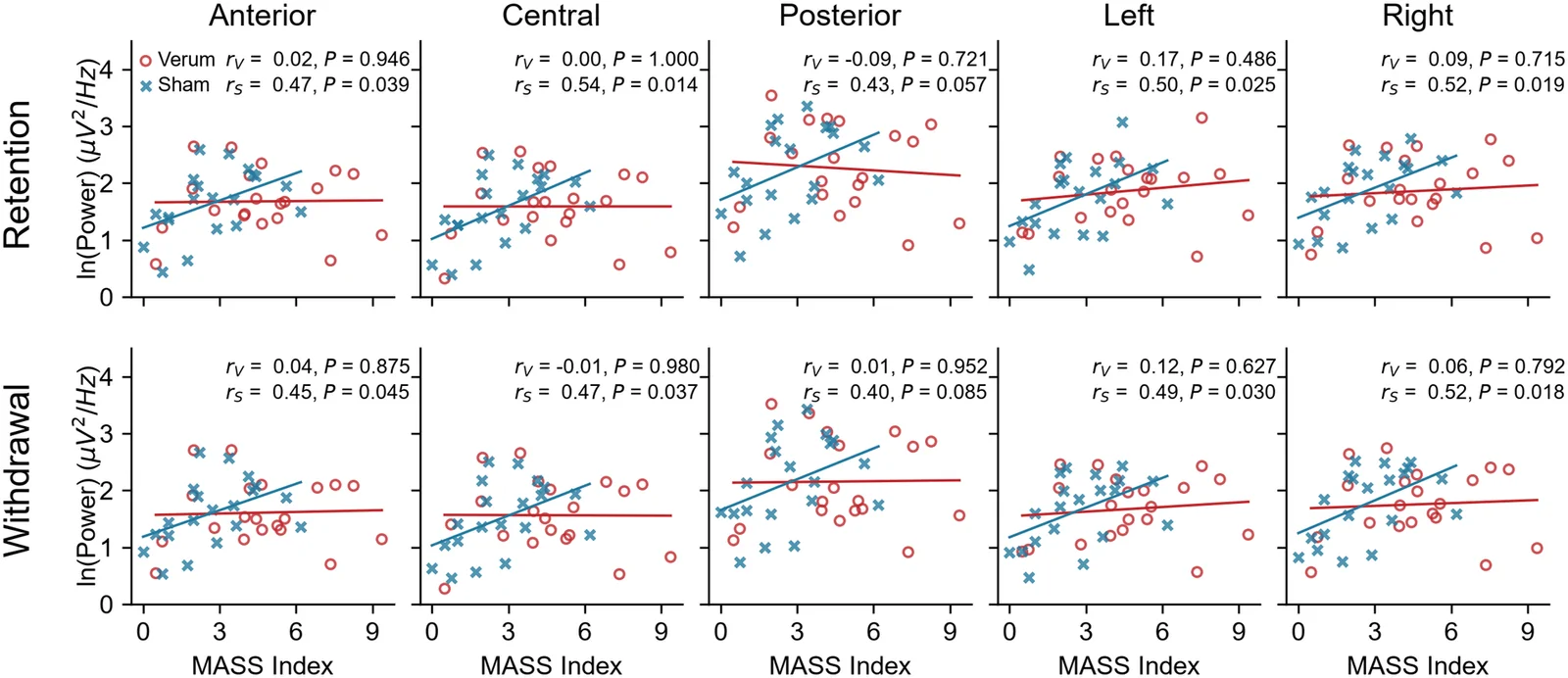
Correlation between MI and beta AP.

## Discussion

Since the clinical effects of non-penetrating sham acupuncture were recognized, mechanistic studies have evolved from demonstrating that it elicits BOLD activity similar to verum acupuncture (16) to advancing the view that sham acupuncture may involve cognitive components comparable to those of verum acupuncture (9). Our study adds electrophysiological evidence to this growing body of work. In contrast to studies focusing on visual stimulation (17) or mental imagery (18) related to acupuncture procedures, the current findings emphasize that non-penetrating skin touch at acupoints and the associated sensory attention alone can elicit electrophysiological activity similar to, though partially weaker than, that of verum acupuncture. These effects, including changes in theta, alpha, and beta band power, emerged during the clinically relevant needle retention phase and persisted into the post-withdrawal phase, suggesting sustained neural modulation and lasting aftereffects.

The increase in theta power observed over the central region may reflect nociceptive sensation and attentional processes. Previous research has linked acupuncture to nociceptive sensation, and cognitive regulation, demonstrating that both deqi elicited by needling at acupoints and pain evoked by non-acupoint can rapidly modulate the efficiency of attention networks in healthy individuals (33). Furthermore, converging evidence links theta oscillations to acupuncture, somatosensory stimulation, pain processing, and attention. For instance, low-frequency transcutaneous electrical acupoint stimulation delivered bilaterally at LI4 has been shown to increase theta power in the parahippocampal gyrus and decrease alpha power in the postcentral gyrus within 10 min after stimulation onset (34). Elevated theta and beta activities have been observed in patients with chronic pain (35), and enhanced theta oscillations in the primary somatosensory cortex have been documented in rat models of both acute and chronic pain (36). Additionally, increased fronto-central theta activity is associated with selective attention allocation (37) and somatosensory discrimination (38). Collectively, this evidence supports our interpretation that the increased central theta power reflects a central pathway in which acupuncture, including non-penetrating sham acupuncture, acts as a salient somatic input that captures attention.

Beta oscillations are known to play a role in sensorimotor processing (39). The beta-band increase observed in the present study may be interpreted in light of the widely accepted “status quo” hypothesis (32). Specifically, the instruction to focus on bodily sensations and together with the concurrent increase in theta activity, suggests engagement of endogenous top-down processes. This active maintenance of the current cognitive set is known to require enhanced beta activity. The increase in beta power may index attentional resource allocation, consistent with prior reports of a negative association between fronto-central beta power and inattention scores (40).

The reduction in alpha power during the post-withdrawal phase relative to the retention phase was unexpected, as previous studies had reported that acupuncture on LI4 (41) and ST36 (42) induced a significant and sustained increase in alpha power. For the current results, there might be the following potential explanations. Unlike previous studies, the participants were explicitly instructed to focus on the sensations experienced, which might lead to a group-level effect and enhance the components of information processing. Alpha/beta power decreases parametrically track the fidelity of both externally-presented and internally-generated stimulus-specific information represented within the cortex (43). The alpha power might be influenced by both the external acupuncture stimulation and the internal information processing during retention phase, while it is dominated by the latter one during post-withdrawal phase. This internal information processing might also be related to the residual sensation during post-withdrawal phase. Previous research has linked the reduced alpha power to the reporting of tactile sensations in the absence of actual stimuli (44). Under this interpretation, post-acupuncture residual sensations may not arise solely from peripheral physiological effects. Similar phenomena may also be expected in acupuncture-imagery paradigms that elicit deqi sensations. Further investigation is warranted to clarify the underlying mechanisms.

At this point, we have discussed the potential cognitive explanations for the similar electrophysiological effects induced by verum and sham acupuncture. These cognitive processes arise from the sensory experiences of verum and sham acupuncture on the body surface. Despite the reduced somatosensory input of sham acupuncture compared to verum acupuncture, the cognitive aspects of sham procedures may closely resemble those of actual acupuncture treatments, and this has already attracted the attention of researchers in the field (9, 45, 46). The current research results provide new electrophysiological evidence for these discussions.

Finally, the exploratory correlation analysis offered an additional lens through which to interpret the associations between sensations elicited by verum versus sham acupuncture and EEG activity. Although the overall effects appeared similar, the underlying neural processes may differ. Across widespread scalp regions, deqi sensations elicited by verum versus sham acupuncture showed distinct associations with theta and beta activity. The observed negative association between theta power and self-reported deqi intensity may be partially explained by recent study (47). In that study, lower intrinsic attention to pain was associated with higher theta power within the ascending nociceptive pathway and the default mode network during moderate-intensity pain evoked by transcutaneous electrical nerve stimulation. When superficial stimulation reaches sufficient intensity, as observed in the verum but not the sham condition, this negative association may reflect individual differences in intrinsic attention to nociceptive sensation. Such variability may arise from stable traits factors or may represent an adaptive response to pain, whereby thalamic bursts firing could contribute to cortical theta desynchronization and a reduction in sensory salience (48). This mechanism may also help explain why the relationship between deqi sensations and beta activity was attenuated under verum acupuncture yet remained evident under sham acupuncture.

Collectively, these findings suggest that both verum and sham acupuncture, through cutaneous stimulation and associated attentional engagement, may initiate a series of cognitive processes. Although these effects have yet to be fully elucidated, the current results indicate that the non-penetrating components of acupuncture are not merely passive background conditions; rather, they constitute active mechanisms capable of eliciting measurable central responses. These mechanisms may represent one potential component of acupuncture's therapeutic effects. Accordingly, our findings provide empirical evidence that helps account for the “non-specific effects” of acupuncture and offer a new perspective for re-evaluating the relationship between outcomes following verum versus sham acupuncture. It should be noted that the aim of this study was not to investigate acupoint specificity or clinical efficacy, and that the sample consisted of only 20 healthy, acupuncture-naive participants. Therefore, caution must be exercised when generalizing the findings to specific clinical settings. Future studies involving broader populations and employing more advanced methods such as deep learning, may help to further elucidate the mechanism of the effect of non-penetrating sham acupuncture.

## Conclusion

The current study indicates that non-penetrating tactile stimulation at acupoints, together with the accompanying sensory attention, can evoke electrophysiological responses similar to, though partially weaker than those observed with verum acupuncture. These responses demonstrate distinct temporal dynamics and may be associated with specific psychological processes, such as somatosensory processing and attentional allocation, that could plausibly contribute to therapeutic effects.

## Data Availability

The original contributions presented in the study are included in the article/Supplementary Material, further inquiries can be directed to the corresponding author.
